# Burial Customs on the Continent

**Published:** 1882-03

**Authors:** 


### BURIAL CUSTOMS ON THE CONTINENT.

In France, as most people are aware, no one meeting a funeral on the streets omits to raise the hat or cap in token of respect; but in Spain this usage does not exist. When the "Viatique" is carried through the streets, every one is bare-headed and kneeling, but a funeral passing along receives no mark of respect as in France. Moreover, while in the latter country a deceased person is followed to the cemetery by all his relatives, friends, acquaintances, and even by many who are only acquaintances of his acquaintances, in Spain it is the habit for persons to abstain from accompanying the coffin to the grave. If the defunct belongs to the better classes, his friends send their carriages to follow, but they themselves remain at home. The Spanish cemeteries differ also materially from those in France. "They are," said M. Emile Maison, who has resided many years at Madrid, "but walls provided with drawers, only a few monuments being seen in the enclosure, erected to the memory of the wealthy or distinguished."

To leave Spain and its customs and halt on Italy's classic soil, there are one or two things worth mentioning in reference to the burial of the dead, which is performed with a different ceremonial in different parts of the country. One remark which applies to the whole of Italy, may be made, however, namely, that the hearse is entirely unknown. *Apropos* of the hearse, its introduction into France only dates from Louis XIV's time; and when it was first used to carry the dead to the cemetery, the innovation was loudly condemned by the public. At Turin the interment of the higher classes takes place generally at dusk; the followers are numerous, but are mostly composed of valets or servants of the friends or relatives of the deceased, clad in rich liveries for the occasion.

At Naples funeral ceremonies are conducted with a certain parade and pomp. The dead man, woman, or child is exhibited, richly dressed, on the bed. Sometimes, indeed, the body is thus exposed to view under the porch of the house, surrounded with lighted tapers and flowers. When the moment arrives for placing it on the bier the duty is discharged by a religious community, excepting in the case of the poor, whose remains, as in France, are consigned to the "fosse commune," which is, in fact, nothing but a deep well. In the magnificent Neapolitan cemetery, which forms an amphitheatre, there are 365 of these wells, one for every day in the year. Every day one of them is opened to receive the dead, a quantity of quicklime is emptied into it, a few pails of water are poured on and the stone is replaced, to be removed again only at the expiration of a twelvemonth. This is how the remains of the poorer classes are disposed of. With regard to the wealthier portion of the community they are interred in a monument resembling a chapel. The coffin is not lowered into a vault, for the reason that there are none, but is placed in the chapel itself, and covered with a slight layer of prepared earth, which has the property of reducing the body to a skeleton within a year from the date of interment. The family of the deceased person then proceed with another funeral ceremony. The bones are collected, put into a fresh coffin of peculiar shape and walled up, the name and quality of the defunct being inscribed on the stone which shuts in the coffin.

At Palermo the dead are placed in a bier richly covered with red gilt embroidered velvet, or in a kind of sedan chair equally red, and conveyed to the convent. On its arrival the body, after the funeral service has been performed, is lowered into a large "souterrain," which extends under the convent gardens. Here the uncoffined remains are placed in a vault, the ground of which is formed of extremely fine sand. Each receptacle is made to hold six or eight corpses. It is called the Scolatojo, and when filled is walled up for a year.—*Churchman's Shilling Magazine.*

---

God is the only being who has time enough; but a prudent man, who knows how to seize occasions, can commonly make a shift to find as much as he needs.—*Lowell.*

Wave lengths of the sounds emitted by a man's voice in ordinary conversation are from eight to twelve feet, and that of a woman's voice two to four feet per second.

The friction of two bodies, one against another, produces heat. By rubbing together two pieces of ice in a vacuum below zero, Sir H. Davy partially melted them.

There were sanitarians in the days of our ancestors. So long as eight hundred years ago, in the time of Richard II., an ordinance was enacted forbidding the pollution of rivers, drains, etc.; another in the reign of Edward II. against selling "muzzled swine-flesh," etc.; and in the reigns of Henry VI. and Henry II. and Elizabeth, for the inspection and cleansing of sewers, against the slaughtering of cattle in towns, and against the overcrowding of dwellings.

